# The Effect of Autoregulated Flywheel and Traditional Strength Training on Training Load Progression and Motor Skill Performance in Youth Athletes

**DOI:** 10.3390/ijerph18073479

**Published:** 2021-03-27

**Authors:** Niklas Westblad, Henrik Petré, Andreas Kårström, Niklas Psilander, Glenn Björklund

**Affiliations:** 1 Department of Physiology, Nutrition and Biomechanics, The Swedish School of Sport and Health Sciences, 114 33 Stockholm, Sweden; henrik.petre@gih.se (H.P.); niklas.psilander@gih.se (N.P.); 2 Swedish Winter Sports Research Centre, Department of Health Sciences, Mid Sweden University, 831 25 Östersund, Sweden; Andreas.karstrom@miun.se (A.K.); glenn.bjorklund@miun.se (G.B.)

**Keywords:** countermovement jump, peak height velocity, sprint, squat jump, resistance training, autoregulation

## Abstract

Background: The effects of flywheel resistance training (FRT) on youth are relatively unknown. The aim of this study was to compare the effects of autoregulated FRT with traditional strength training (TST) on jumping, running performance and resistance training load progression in youth athletes. Thirty youth athletes (11.8 ± 0.9 yr) were matched for peak height velocity (PHV) status and block-randomised into two groups: FRT (*n* = 15, PHV −0.8 ± 1.6) and TST (*n* = 15, PHV −0.8 ± 1.5). Twelve resistance training sessions over a six-week intervention with flywheel or barbell squats were performed using autoregulated load prescription. Squat jump (SJ); countermovement jump (CMJ); and 10 m, 20 m and 30 m sprints were assessed pre- and post-intervention. The external load increased similarly for FRT and TST (*z* = 3.8, *p* = 0.06). SJ increased for both groups (*p* < 0.05) but running performance was unaffected (*p* > 0.05). Conclusions: FRT resulted in similar load progression and motor skill development in youth athletes as TST, but the perceived exertion was less. Autoregulation is a practical method for adjusting training load during FRT and should be considered as an alternative to autoregulated TST.

## 1. Introduction

Fundamental motor skills (FMS), such as running and jumping, promote physical activity and increase athletic performance in youth [1]. Strength development is a critical component for the successful development of FMS [2]. Methods such as traditional strength training (TST) with free weights, plyometric training (PT) or a combination of TST and PT have been shown to increase muscular strength and motor skills in youth athletes (physically active youth 6–18 years of age) [3,4,5,6]. An alternative resistance training method that has increased in popularity, yet is not well studied in youth athletes, is flywheel resistance training (FRT). FRT relies on resistance created by inertia instead of traditional gravity-dependent strength training (TST with free weights). Resistance training with a flywheel device enables a variable resistance where the load mainly depends on the number of flywheels, the size of the flywheels and the effort exerted by the athlete to put the flywheel into motion [7,8]. The flywheel will only resist the force acted upon it, which means that the amount of resistance is completely dependent upon the effort exerted by the performing athlete. FRT is therefore safer than TST as the athlete fatigues because it reduces the risk of overload and injury, especially as the harness used during the FRT has been shown to potentially reduce the compression force on the lumbar vertebrae area, as compared to TST with barbell [9]. This is particularly important during periods when the overall training volume is high. FRT is also a more practical and time-efficient training modality as the equipment is compact and mobile compared to TST and does not require loading/unloading of weights. In addition, variable resistance has been shown to induce greater training stimulus than TST in youth [10]. 

FRT has been shown to elicit performance enhancements in squat jumps (SJ) [11,12,13], countermovement jumps (CMJ) [11,13,14,15,16,17] and sprint times [12,14,16,18] in adult participants. Likewise, FRT has been proven to increase maximal strength and power similar to TST [19]. However, relatively little is known about how FRT influences FMS in youth. Intensity is a key variable during resistance training. The most common method for prescribing intensity during a resistance exercise is by using an intensity based on an athlete’s pre-determined 1-repetition maximum (1RM). A previous study suggested that an exercise load between 80 and 89% of the 1RM is suitable for increasing muscle strength in youth athletes [5]. The problem with this method is that it does not consider individual readiness for a particular training session, which is a limitation due to inaccuracies. There is an intrinsic, intra-individual variation in the rate of progression and recovery in skeletal muscle exposed to resistance training [20]. To base the intensity on a pre-determined 1RM could increase the risk of a mismatch between induced fatigue and optimal fitness adaptations. For example, if the load is too low for a training session due to a fast rate of recovery and progression, the stimuli for adaptations will be sub-optimal. If the load is too high, there is a risk of hampered physiological adaptations due to overreaching or injury. In addition, the athlete’s training background has been shown to affect the validity of this method, whereby endurance athletes can complete significantly more repetitions at the pre-determined 70% and 80% of 1RM compared to that of weightlifters [21].

Resistance training prescriptions, with frequent adjustments based on feedback from athletes (autoregulation), could provide a better option for strength development [22,23,24]. A practical way to adjust the training intensity based on autoregulation is by using a subjective rating scale, such as a 1–10 rating of the perceived exertion after each set in a training session, set-rating of perceived exertion (set-RPE), and adjusting it directly before performing the next set. Autoregulation using a 1–10 rating scale after each set has been used successfully when regulating resistance during traditional free-weight resistance training in female youth athletes [25]. For FRT, autoregulation using a 1–10 set-RPE scale has been used during single training sessions to prescribe training intensity when measuring contractile function post exercise in adults [26] and with a 6–20 scale to prescribe intensity in youth soccer players during a training intervention [27]. However, so far, no study has compared the effects of autoregulated FRT with autoregulated TST.

In summary, there is a lack of studies investigating the use of subjective autoregulation with a 1–10 set-RPE scale during FRT in youth, nor are there an adequate number of studies investigating the effect of FRT on FMS in youth [27]. The primary aim of this study was to examine the effect of two autoregulated resistance training methods (FRT compared to TST) on load progression, jumping and running performance in youth athletes.

## 2. Materials and Methods

### 2.1. Participants

**Figure 1:**
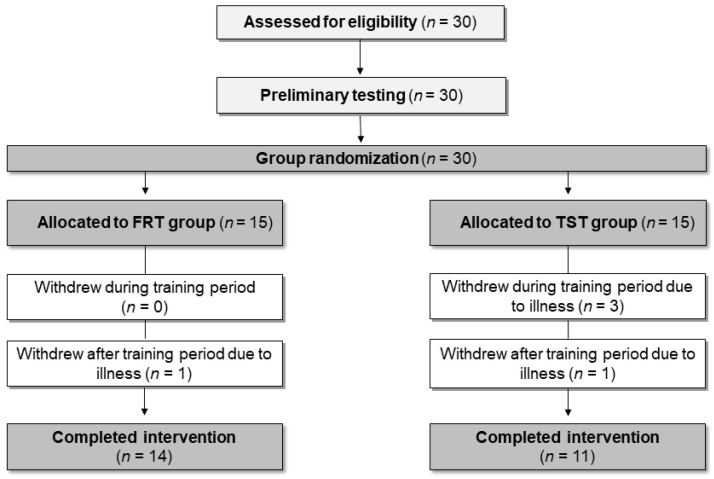
Flow chart of the participant’s progress from eligibility assessment to completion of intervention.

Thirty healthy and physically active youth (11.8 ± 0.9 yr; 155.2 ± 8.3 cm; 44.5 ± 9.6 kg; *n* = 15 boys and *n* = 15 girls) from an athletics club volunteered to participate in the study, and twenty-five completed the study (Figure 1). The participants were physically active at least twice a week at the athletics club but were unfamiliar with resistance training. All participants and their parents were informed about the purpose and risks of participating in the study. Participants and their parents provided written informed consent. The study was pre-approved by a Swedish regional ethical review board, DNR 2018/765-31/1, in accordance with the Declaration of Helsinki.

### 2.2. Experimental Design Overview

The participants were matched for peak height velocity (PHV) status and block-randomised into two groups: FRT (*n* = 15, time to PHV = −0.8 ± 1.6) and TST (*n* = 15, time to PHV = −0.8 ± 1.5) (Table 1). Data on maturity statuses were presented for each group, as is recommended for paediatric research [6]. To assess the vertical jumping ability, performance tests of CMJ, SJ and sprinting ability with 10 m acceleration, 20 m flying sprint and 30 m sprint, were conducted before and after the training period. All tests were performed in a rested state pre- and post-intervention. Twice a week for 6 weeks, both training groups performed their training mode in addition to their regular training, which consisted of 1–3 sessions per week of track and field sessions including a mixture of sprinting hurdles, long jumps and shot put.

### 2.3. Testing and Familiarisation

Standing and seated height (centimetres) of all participants were measured using a stadiometer attached to a wall. Body mass (kilograms) was assessed using an electronic scale (FitScan BC-545F, Tanita Corporation of America). The average of three assessments was set as the participant’s body mass in kilograms (Table 1). Anthropometric data were used to estimate the biological maturational status pre- or post-PHV and it was calculated using the equations proposed by Mirwald et al. [28].

To familiarise the participants with the testing and training procedures, three familiarisation sessions consisting of three sets of 10 bilateral squats with a flywheel device (kBox 4 Pro, Exxentric AB, Sweden) and three sets of 10 bilateral squats with a 10- to 20-kg barbell were performed by all participants before randomisation. Three sessions of FRT have been used in previous studies to familiarise participants with flywheel training and to minimise effects of learning [18]. Participants were instructed to perform a full-depth squat with the femur trochanter major positioned lower than the lateral femur epicondyle. Individual feedback was given if a participant could not perform a correct squat movement.

At least 48 h after completing the last familiarisation session, all participants performed functional neuromuscular performance tests in the following order: CMJ without arm-swing, SJ and a 30 m sprint (including 10 m and 20 m splits). To ensure test–retest validity and reliability, a test official (N.P. or H.P.) supervised the same assessment pre- and post-test. The CMJ and SJ height were recorded using a photocell system that calculates the jumping height out of airtime (IVAR Jump and Speed Analyser, LN Sport Consult, Sweden). In the CMJ, participants were instructed to perform a vertical jump from a squatting depth of their own choice with encouragement to perform the jump as fast as possible to maximise jump height. The SJ was performed from a half-squat position with a 90° knee angle from a 2 s isometric start before performing a vertical jump [29]. For both tests, participants were instructed to hold their hands akimbo during the entire jump and then land with a rebound jump to naturally land on their toes with a straight leg. Each participant performed three attempts each for CMJ and SJ with a one-minute rest between each attempt. Their best attempt was selected for further analysis.

Sprint times were recorded using wireless timing gates (Brower TC Timing System, Draper, UT, USA) with single-beamed photocells. Sprint times were acquired from photocells placed at 10 m and 30 m with a stationary start 0.3 (m) behind the first timing gate. The flying 20 metre sprint time was calculated as the 30 m time minus the 10 m time. Starting timing gates were placed at a low height (0.6 m) to reduce the chance of a measurement error by preventing legs and arms from triggering the photocell [30]. Each participant performed three attempts with a one-minute rest between each attempt, and the best attempt was selected for further analysis.

### 2.4. Training Intervention

**Figure 2:**
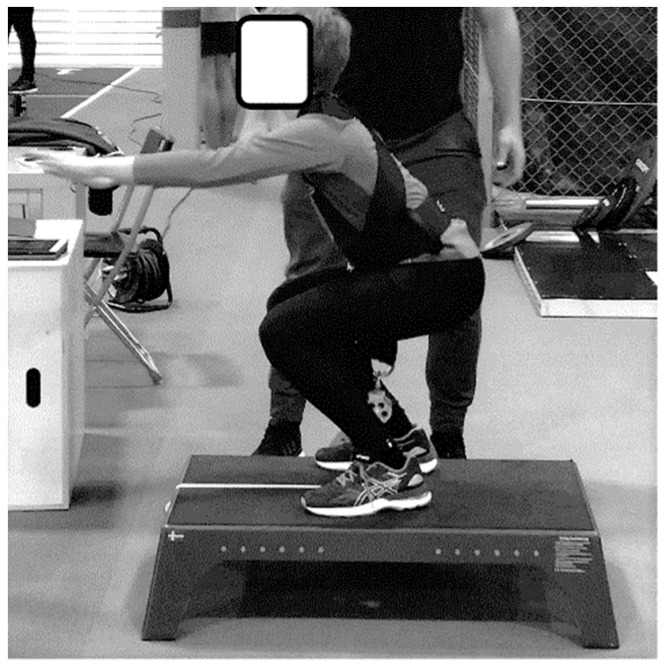
A squat performed on the flywheel device.

Resistance training was performed twice a week for 6 weeks. A minimum of 48 h rest was required between each strength session to allow participants to adequately recover. Fully qualified trainers (N.P. and H.P.) supervised all training sessions and every repetition performed by all participants to ensure correct technical execution and exercise intensity. The intensity of the exercise was increased only if the technical execution was satisfactory. If the technical squat movement was not performed correctly, the intensity was decreased or the participant received individual technical feedback on how to perform the movement properly. Each session started with a 10 min standardised aerobic warm-up, which consisted of either running or cycling on a bicycle ergometer. Training sessions lasted no longer than 30 min with 2 min inter-set rest periods. Both groups performed the squat exercise in four sets of six repetitions on either the flywheel machine (Figure 2) or using a free-weight barbell. The FRT group performed two submaximal pre-repetitions at low intensity to initiate the rotational force of the flywheel. The FRT group started with a 0.025 kg·m^2^ flywheel, and the TST group started with a load of 10–30 kg, depending on what the trainer decided was adequate. During the eccentric phase of the movement, both groups were instructed to resist with full control and evenly through full range of motion.

### 2.5. Autoregulation (set-RPE)

Before and during the training intervention, all participants were verbally informed about the autoregulation method so that they fully understood the rating procedure. After each set, participants were asked to self-report their effort on a modified scale of 1 to 10 by Hackett et al. [31], with lower values representing lower effort and higher values representing higher effort (set-RPE). RPE is a valid method for assessing and prescribing the intensity of resistance training [31]. The supervising trainers set the exercise load for both training groups to match a set-RPE of 8 by adjusting the load on the barbell for the TST group or by encouraging the participant to increase or decrease the speed of movement or adjust inertia (more/less flywheels) during FRT. Training resistance ranged from 10 to 75 kg for TST and from 0.25 to 0.05 kg·m^2^ for the FRT group.

### 2.6. Statistical Analysis

All data were checked for normal distribution using the Shapiro–Wilk test. Values were reported as the mean ± SD for physical characteristics and performance variables. Set-RPE and training data are presented as median and interquartile range (IQR), as they were not normally distributed. Statistical analyses were performed using Jamovi [32]. An unpaired *t*-test was used to perform comparisons between FRT and TST to record pre-intervention physical characteristics. The effect size was determined using Hedges’ *g* with weighted standard deviation. Comparisons for all performance variables were analysed using 2 × 2 (group × time) repeated measures two-way analysis of variance (ANOVA), where groups represent FRT and TST, and time represents pre- to post-training data. The data sphericity was tested by Mauchly’s test of sphericity and was not violated. Generalised Eta squared (η^2^_G_) was calculated and used as the effect size to determine the meaningfulness of the differences in ANOVA. Generalised Eta squared was interpreted as small η^2^_G_ < 0.02, medium η^2^_G_ = 0.13 and large η^2^_G_ > 0.26 [33]. The calculated percentage of change in training load included all sets and was presented as the peak power average (FRT) or lifted weight in kilograms (TST) for the first ten sessions, which was the limit of compliance that needed to be fulfilled for inclusion in the following analysis. A Mann–Whitney *U* test was used for overall group comparisons between FRT and TST for set-RPE and percent development using the point biserial correlation coefficient (*r*_pb_) as the effect size. Interpretive benchmarks were small *r*_pb_ < 0.10, medium *r*_pb_ = 0.24 and large *r*_pb_ > 0.37 [34]. Moreover, a comparison between the training data was tested with Friedman’s test. An alpha level of 0.05 was set a priori.

## 3. Results

### 3.1. Training Compliance

Five participants (FRT *n* = 1 and TST *n* = 4) dropped out during the intervention due to illness. The FRT group completed a mean of 96% of the total training sessions, and the TST group performed a mean of 95% of the total training sessions. There was no difference in training compliance between FRT and TST (z = 0.08, *p* = 0.94, *r*_pb_ < 0.01). The minimum amount of training sessions completed for inclusion was set to 80% (10 out of 12 training sessions). Body mass increased pre to post (*F*_1,23_ = 15.4, *p* < 0.001, η^2^_G_ < 0.02: small) with no difference between groups (*F*_1,23_ = 0.56, *p* = 0.46, η^2^_G_ = 0.02: small) or interaction effects (*F*_1,23_ = 0.78, *p* = 0.385, η^2^_G_ < 0.02: small).

### 3.2. Training Load and Set-RPE

**Figure 3:**
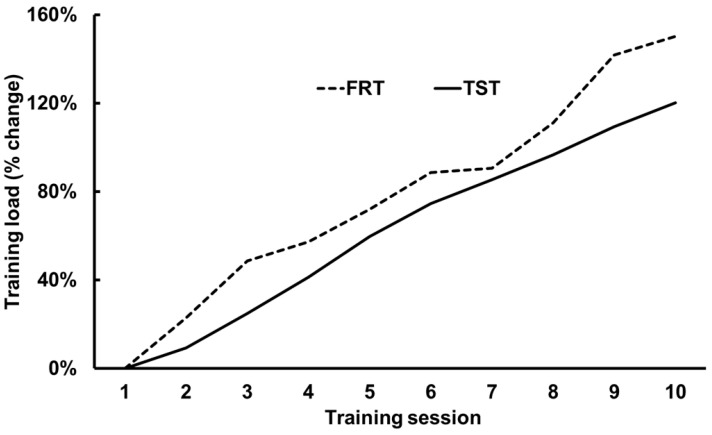
Change in average peak power produced (W) and weights (kg) lifted during the first 10 resistance training sessions with FRT or TST.

The overall percent of development in the training load was similar between FRT and TST (*z* = −0.81, *p* = 0.55, *r*_pb_ = 0.17), with the group average power watts for FRT increasing by 150%, from 53 watts to 111 watts versus TST, which increased the group average for weight lifted by 120%, from 23 kg to 50 kg from the first to tenth training session (*χ^2^*_9_ = 71.4, *p* < 0.001) (Figure 3). The overall set-RPE during the training period was lower for the FRT group compared to that of the TST group (median = 6, IQR = 5–7 and median = 7, IQR = 6–8, respectively) (z = −10.5, *p* < 0.001, *r*_pb_ = 0.37). Throughout the training period, the set-RPE remained unchanged from start to finish (*χ*^2^_9_ = 16.6, *p* < 0.06).

### 3.3. Sprint and Jump Characteristics

Pre- and post-intervention sprint and jump data are presented in Table 2. Training intervention had a minor influence on sprint performance, with a trend for improvement from pre- to post-intervention for 10 m acceleration (s) (*p* = 0.08, η^2^_G_: medium). There were no differences between the groups (*p* = 0.45, η^2^_G_: medium) or interaction effects (*p* = 0.60, η^2^_G_: small). The 20 m flying sprint (s) test showed no difference over time (*p* = 0.94, η^2^_G_: small), but there was a meaningful difference between the groups (*p* = 0.11, η^2^_G_: large) with no interaction for group × time (*p* = 0.53, η^2^_G_: small). For the 30 m sprint (s), there was a non-significant medium effect between groups (*p* = 0.13, η^2^_G_: medium), but not for time or interaction (*p* = 0.36 and *p* = 0.88, both η^2^_G_: small). Over time, SJ (cm) improved (*p* = 0.01, η^2^_G_: medium), but CMJ (cm) did not (*p* = 0.40, η^2^_G_: small). There were no group differences regarding SJ (cm) or CMJ (cm) (*p* = 0.65, η^2^_G_: small and *p* = 0.47, η^2^_G_: small, respectively) or interaction effects (*p* = 0.51, η^2^_G_: small and *p* = 0.77, η^2^_G_: small, respectively).

## 4. Discussion

To our knowledge, this is the first study comparing the effects of autoregulated FRT with TST on load progression, jumping and running performance in youth athletes. Our findings demonstrate that autoregulated FRT and TST improve jumping but not running performance and that both training modalities achieve similar increments in training load progression. Although no significant effects on sprint performance were observed, it is likely that some individuals experienced improvements in 10 metre acceleration, as the results for both groups showed a trend towards a significant effect.

The present results of FRT on sprint performance are in contrast with a recently published study on FRT by Fiorilli et al. [27], but is in line with findings by Raya-González et al. [35]. Both studies included youth soccer players performing FRT in addition to their regular soccer training programme. Fiorilli et al. [27] showed increased performance in the 60 m linear sprint after 6 weeks of FRT, two times per week, whereas Raya-González et al. [35] showed no increments in acceleration or linear sprint performance (10, 20 and 30 m sprint) after ten weeks of FRT, one session per week. Interestingly, the participants in both studies improved in vertical jump performance (SJ and CMJ for the study by Fiorilli et al. and Raya-González et al., respectively) [27,35]. One important difference between these studies and the present study is the direction of the applied load during FRT. In the study by Fiorilli et al., resistance was applied in a horizontal direction to simulate sprint starts [27]. In the study by Raya-González et al. [35], the participants performed lateral squats in the horizontal direction. It is well known that movement-specific strength training increases the transfer of maximal strength to explosive performance [36], and this could make up for the different effect that FRT has on sprint performance. Further support for this theory is that Raya-González et al. [35] observed improvements in the change of direction performance, which highly mimics movement during lateral squat FRT. Interestingly, even though resistance was applied in a horizontal direction during training, soccer players in the study by Fiorilli et al. [27] significantly increased their jump height performance during SJ, which demonstrates that an assumption of specificity is not always valid.

The present results from autoregulated TST are in line with those in a previously published study by Arede et al. [25], which showed a significantly positive effect on SJ but no significant effect on 10 and 20 m sprint performance in female youth basketball players. However, in contrast to our findings, Arede et al. [25] showed improvements in CMJ that could be explained by differences in the duration of interventions (8 weeks compared to 6 weeks) and in the inherent movement specificity in regular training (track and field training compared to basketball training) which was performed parallel to the interventions. Basketball involves explosive plyometric-specific training in a vertical direction, whereas most track and field training does not. Plyometric exercises require the neuromuscular system to involve a stretch shortening cycle, which is used in explosive movements such as CMJ but not SJ [37]. Furthermore, participants in the present study were unfamiliar with resistance training compared with basketball players in the study by Arede et al. [25], so good technique rather than high movement velocity was prioritised during training. To summarise, both FRT and TST need to be highly specific to assure increased performance in sprint and jump performance during a 6 to 10 week training period in youth athletes. This assumption is supported by a recently published umbrella review highlighting the principle of training specificity in youth during resistance training [4].

To our surprise, we discovered that the flywheel training group rated an entire set-RPE lower than the TST group, despite similar progression in training load. This might be explained by the difference in resistance (variable load during FRT compared with constant load during TST). During squats with TST, the movement starts and ends with straight hips and legs, and the intensity is set with weights on the barbell. During squats with FRT, the movement starts and ends with flexed hips and legs, and the load is adjusted with effort from the participant during the concentric phase of the movement (transfer of energy to the flywheel). This means that during TST, the participants had no choice but to exert the intensity needed to lift the weight in order to complete the repetition, which was not the case during FRT. Self-regulating FRT could therefore potentially be perceived as easier than TST, even though the load is similar. Another potential reason for the difference in the total RPE rating is that it might take more sets to determine the right autoregulated intensity during FRT compared with TST.

Despite the practical novelty of the present findings, this study included some limitations. First, the rapid increase in training load that was observed for the two groups might be explained, in part, by the participant’s limited experience with resistance training. Although familiarisation sessions were implemented as part of the study following recommendations [18], these recommendations are mainly valid for an adult population. Secondly, the lack of a maximal strength test (1RM-test) makes it difficult to draw any certain conclusions on how the training protocols affected strength development. The reason for not including such a test or a multi-repetition maximum test was that we did not find it suitable for our young participants, who were unfamiliar with resistance training. The 1RM tests demand a certain skill level to measure the true lower-body strength [38] and to be safe. In addition, performing 1RM tests at single time points does not consider inaccuracies in terms of balance between recovery and progression, which is highly individual. Therefore, we believe that monitoring the increments in training load during an intervention is a better choice for assessing strength increments than implementing pre- and post-1RM tests. Finally, findings from this study should be interpreted with caution due to the relatively low number of participants. More studies are needed to confirm our findings. Hence, we suggest that future studies investigating FRT include a larger sample size, youths who are more familiar with resistance training and 1RM measurements.

## 5. Conclusions

This is the first study to examine the effects of autoregulated FRT on training load progression, running and jumping performance in youth. FRT resulted in similar training load progression and motor skill development in youth athletes as compared to traditional strength training, but the perceive exertion was less. The results show that FRT and TST improves jumping but not running performance, highlighting the need of training specificity to assure increased transfer to functional performance. Autoregulation is a practical and effective method for adjusting the training load during FRT. It should therfore be considered as an alternative to autoregulated TST during periods/situations when TST is not optimal; for example, during the in-season period when the overall training load is higher and strength needs to be maintained in a safe manner to avoid injuries and ensure long term development.

## Figures and Tables

**Table 1:** Physical characteristics pre-intervention.

Variable	FRT (*n* = 14)	TST (*n* = 11)	*p* Value (95% CI)	Effect Size
Height (cm)	155 ± 8	156 ± 9	*p* = 0.83 (−7.78 to 6.33)	*g* = 0.11
Body mass (kg)	43.1 ± 9.0	46.3 ± 10.4	*p* = 0.41 (−11.29 to 4.77)	*g* = 0.32
Age at PHV (yr)	13.1 ± 1.3	13.2 ± 1.1	*p* = 0.85 (−1.09 to 0.90)	*g* = 0.08

FRT, flywheel resistance training; TST, traditional strength training; PHV, peak height velocity; CI, confidence interval. Effect size is expressed as Hedges’ *g*. Data are presented as mean ± SD.

**Table 2:** Sprint and jump characteristics for both training groups.

Variable	Time	FRT	TST	*F* = Value	*p* = Value	η^2^_G_
10 m acceleration(s)	Pre	2.15 ± 0.10	2.19 ± 0.12	=^a^ 3.43	=0.08	0.03
	Post	2.12 ± 0.11	2.14 ± 0.16	=^b^ 0.59	=0.45	0.02
				=^c^ 0.28	=0.60	<0.01
20 m flying sprint(s)	Pre	2.96 ± 0.15	3.09 ± 0.26	=^a^ 0.01	=0.94	0.00
	Post	2.95 ± 0.18	3.10 ± 0.27	=^b^ 2.78	=0.11	0.10
				=^c^ 0.40	=0.53	0.01
30 m sprint(s)	Pre	5.12 ± 0.24	5.31 ± 0.37	=^a^ 0.88	=0.36	0.01
	Post	5.10 ± 0.26	5.29 ± 0.37	=^b^ 2.45	=0.13	0.09
				=^c^ 0.02	=0.88	0.00
SJ (cm)	Pre	23.4 ± 5.0	22.6 ± 4.8	=^a^ 7.03	=0.01	0.02
	Post	24.5 ± 5.3 ^#^	24.5 ± 5.7 ^#^	=^b^ 0.21	=0.65	<0.01
				=^c^ 0.51	=0.48	<0.01
CMJ (cm)	Pre	25.5 ± 5.0	24.3 ± 4.9	=^a^ 0.73	=0.40	<0.01
	Post	26.2 ± 4.6	24.6 ± 4.8	=^b^ 0.53	=0.47	0.02
				=^c^ 0.09	=0.77	0.00

The values are presented as mean ± SD. FRT, Flywheel resistance training; TST, traditional strength training; SJ, squat jump; CMJ, countermovement jump. ^a^ Main effect: time; ^b^ Main effect: group; ^c^ Interactive effect: time × group. ^#^ = different from pre-post.

## Data Availability

The data presented in this study are available upon request from the corresponding author.
